# Prevalence, risk factors, and interventions for chronic obstructive pulmonary disease in South Asia: a scoping review protocol

**DOI:** 10.1186/s13643-020-01556-7

**Published:** 2021-01-11

**Authors:** Rafiqul Islam, Md. Tarek Hossain, Nantu Chakma, Afroza Khanom, Tapas Mazumder, Md. Tauhidul Islam

**Affiliations:** International Centre for Diarrheal Disease Research, Dhaka, Bangladesh

**Keywords:** Chronic obstructive pulmonary disease, COPD, Scoping review, Prevalence, Risk factors, Interventions, South Asia

## Abstract

**Background:**

Chronic obstructive pulmonary disease (COPD) is increasingly contributing to the disease burden in South Asia. This review will summarize the prevalence and risk factors of COPD in South Asia and the interventions regarding COPD that have been introduced in South Asian countries.

**Method:**

This scoping review will primarily follow Arksey and O’Malley’s six steps of scoping review methodology. Additionally, it will follow the recent upgradation of the scoping review methodology by Levac et al., and the Joanna Briggs Institute. Research questions were already identified at the beginning of the proposed scoping review. Electronic databases will be searched (PubMed, Web of Science, and ProQuest) using search terms. Studies will be screened independently by two reviewers through a two-stage screening process using pre-developed inclusion criteria for this scoping review. Eligible studies will be abstracted and charted in a standardised form. Preferred Reporting Items for Systematic reviews and Meta-Analyses extension for Scoping Reviews (PRISMA-ScR) will be used to report the result. Additionally, feedback from South Asia’s experienced COPD researchers on the final literature list will be collected for gap identification in literature search. Two independent reviewers will assess the quality of each included study’s design using the Joanna Briggs Institute’s tool.

**Discussion:**

The proposed scoping review will map the evidence on COPD in South Asia through literature review, and it will focus on prevalence, risk factors, and interventions. This review will contribute to the advancement of research on COPD and will be beneficial for policy-makers, public health specialists, and clinicians.

**Supplementary Information:**

The online version contains supplementary material available at 10.1186/s13643-020-01556-7.

## Background

Chronic obstructive pulmonary disease (COPD) is one of the major preventable chronic respiratory diseases (CRD). The Global Initiative for Obstructive Lung Disease (GOLD) defined COPD as a common preventable and treatable disease, characterized by persistent airflow limitation that is typically progressive and related with an enhanced chronic inflammatory response in the airways and the lung to noxious particles or gases [1].

COPD is a leading respiratory disease that deteriorates both the length and quality of lives globally [2]. Like many leading non-communicable diseases (NCDs), COPD is projected to increase in much of the world as the population ages. Globally, the prevalence and death rate of COPD increased by 44.2% and 11.6%, respectively, between 1990 and 2015 [3]. The global burden of disease study estimated that globally there were around 300 million cases of COPD, where it is predicted that there were about 62 million new cases in 2017 [4]. Moreover, the study also estimated that COPD caused 3.19 million deaths in 2017 [5]. It is pertinent to note that, more than 90% of these COPD-related deaths occured in low- and middle-income countries (LMICs) [6]. This could be a result of higher prevalence of smoking and indoor air pollution (IAP) in this region [7]. Alongside, exposure to bacterial and viral infections, various dust, chemicals, vapours, and fumes in the workplace and outdoor air pollution play an imperative role in both the development and progression of COPD in LMICs [2].

South Asia is the most densely populated region of the world and home to almost one-quarter of the world’s population, with a substantial proportion living below the poverty line [8]. Currently, this area of the world is in the midst of an epidemiological transition. In the last 50 years, this region has shown significant improvement in the reduction of premature death and disability from communicable and nutritional diseases such as pneumonia, diarrheal diseases, and malnutrition. On the other hand, NCDs have become the leading cause of death as people are living longer, and globalization and urbanisation are divulging individuals to NCDs risk factors. Concequently, around one-third to two-thirds of the deaths and disability in this part of the world are due to NCDs [9].

According to a report by the World Bank, in 2010, COPD was the second leading cause of death and disability in South Asia [9]. In comparison to the first leading cause of death and disability (cardiovascular diseases), the context of COPD has been under-explored in this region [10]. However, there are some reviews on prevalence [2, 11] and risk factors of COPD [2, 12–15] at the global level, but unfortunately those reviews unable to portray the complete picture of the South Asian region. A similar situation was also observed in the review papers that are particularly focused on interventions for COPD [16–19]. Some review papers on COPD from South Asian countries have been found but most were written for a specific country context such as for Bangladesh [20] and India [21]. The true epidemiology of COPD is important to understand to observe the trends over time and to identify the success or failures of control efforts. Therefore, there is an urgent need to obtain all available data on COPD to accurately gauge the severity of the problem. Understanding the current situation and trends will provide useful insights in the South Asian context that will assist researchers, health professionals, and policy-makers in decision-making; developing future research agendas and strategies; designing and implementing prevention programs to reduce the burden of COPD in this part of the globe.

This scoping review aims to provide a comprehensive understanding of existing literature by reporting COPD rates, and other COPD metrics (e.g., risk factors for COPD; OR with 95% CIs) in South Asian countries (Afghanistan, Bangladesh, Bhutan, India, Maldives, Nepal, Pakistan, and Sri Lanka), by reviewing both published (i.e., in peer-reviewed journals) and unpublished studies (i.e., reports not published in the academic literature). Availability of literature on COPD will be done through a scoping review which, unlike a systematic review [22], offers a much larger perspective in the respective field. Thus, making it a more suitable method to assess the current situation of COPD in South Asia.

## Methodology

**Fig. 1:**
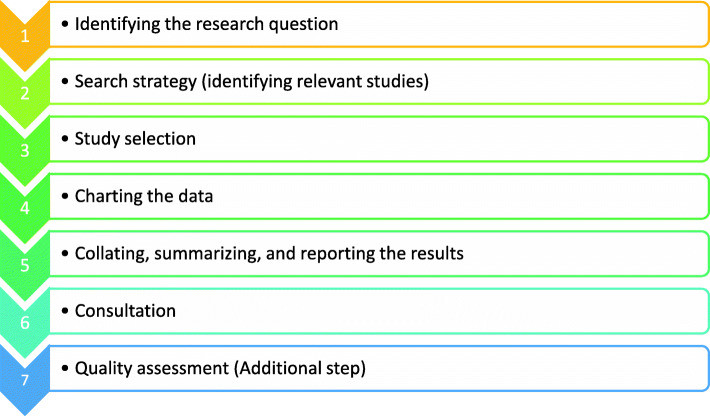
Arksey and O’Malley’s methodological framework with an additional step

There are many different methods for synthesising or reviewing the evidence. Considering the aim of this study, we chose Arksey and O’Malley’s scoping review technique [22] to map the evidence of COPD in South Asia. To understand and convey the breadth and depth of an arena, a scoping review is an increasingly utilized approach for reviewing health research evidence, where the paucity of data makes it difficult to undertake systematic reviews. The six steps of Arksey and O’Malley’s methodological framework are illustrated in Fig. 1. This protocol is in accordance with the PRISMA-Protocols (PRISMA-P) 2015 checklist (Additional file 1: Table S1 for the PRISMA-P 2015 checklist). Suggestions from more recent improvements to Arksey and O’Malley’s framework by Levac et al. [23] and The Joanna Briggs Institute [24] will also be considered during the six steps of review.

Poor quality studies often have a high risk of bias; therefore, the validity of their findings are often questioned by researchers, practitioners, and policy-makers [25]. Therefore, critical appraisal of every study is vital to assess the risk of bias and the reliability of the results. Taking that into consideration,and the recommendations of Levac et al. [23], we decided to include a quality assessment as an additional step in our scoping review. Thus, in seven steps, completion of the scoping review will be done (Fig. 1).

### Step 1: identifying the research question

In this beginning phase, the research team considered various aspects of COPD in South Asia to identify the important issues to be addressed in this scoping review. Subsequqently, defined research questions were developed and linked those questions with the purpose of the study. The final research questions were agreed upon through a series of research team meetings. The research questions were articulated in a way that the readers could clearly understand the scope of inquiry. Through these research questions, we defined the concept, target population, and health outcomes of interest to clarify the focus of this scoping study. Three research questions were identified to guide the scoping review.

- What is the prevalence of COPD in South Asian countries?
- What are the risk factors for developing COPD in South Asian countries?
- What are the interventions introduced in South Asian countries to reduce the burden of COPD?

### Step 2: identifying relevant studies (search strategy)

Eligible studies, both published and unpublished literature relating to our research questions, will be identified in this step. The design of the search strategy was underpinned by the key inclusion criteria (Table 1). These criteria were categorized according to the broad Population—Concept—Context (PCC) mnemonic endorsed by the Joanna Briggs Institute for scoping reviews, which is a less restrictive substitute to the PICO (Population, Intervention, Comparator, and Outcome) mnemonic suggested for systematic reviews. To ensure that the maximum amount of articles and citations related to our research questions are captured, initial exploratory searches in Google and Google Scholar were conducted. Based on the findings of exploratory searches, a broad search term using controlled vocabularies (e.g., MeSH terms) and keywords relating to COPD were generated for searching the literature.

**Table 1:** Inclusion criteria of the proposed scoping review

P	C	C
**Population**	**Concept**	**Context**
Human participants, Any sex, Any socio-economic status	Studies that report the prevalence, risk factors, and interventions regarding COPD or chronic bronchitis.	Research articles conducted in eight South Asian countries (Afghanistan, Bangladesh, Bhutan, India, Maldives, Nepal, Pakistan, Sri Lanka)

Then search terms will be combined using Boolean operators (AND, OR, NOT) to narrow the results. In this review, three electronic databases of the published literature (PubMed, Web of Science, and ProQuest) will be searched to identify the publications. These databases are relevant to public health and accessible. Therefore, it will be helpful for researchers to capture a comprehensive sample of related literature. The proposed search term (Additional file 2: Table S2 for proposed search terms) consisted of the Medical Subject Headings (MeSH) for COPD, prevalence, risk factor, and intervention. In addition, the name of the South Asian countries, other key terms (e.g., community based intervention), and MeSH terms inputted as free text. The search terms might be revised or updated later to obtain the maximum number of articles from the electronic databases before the screening of the articles begins. To ensure that all relevant articles or reports are captured, we will do the backward search of key review articles, snowballing citation and hand searching by using Google and Google Scholar. Further key national journal databases for each country (banglajolinfo [Bangladesh], medindia.net [India], nepjol.info [Nepal], pakmedi.net [Pakistan], sljol.info [Sri Lanka]) will be searched for relevant articles. National journal databases were not found for Afghanistan, Bhutan, and the Maldives. We will also search for a variety of grey literature sources, “Grey Literature Database,” and “clinicaltrial.gov” to identify additional unpublished studies of relevance. We will include all studies related to chronic bronchitis and COPD. However, they will be featured separately in the results section.

### Step 3: study selection

Only original studies written in English will be considered for this review. No limits will be set on publication date, and study design during the selection of the literature. We will not include qualitative studies, as our research questions do not permit us to explore barriers and facilitators for implementing an intervention. Hence, we will limit our study to quantitative studies only. However, review articles including systematic reviews, meta-analyses, meta-syntheses, scoping reviews, narrative reviews, rapid reviews, critical reviews, and integrative reviews will be included in our study. A review of review approach will be applied in this regard but during data extraction, data relevant to South Asian countries and pertaining to our research questions will be extracted only. Both review and primary studies will be identified. During the data extraction process, the data will first be extracted from the review studies, and then the extracted data will be supplemented by the findings from the selected primary studies. During data extraction, primary studies already included in a review will be identified and deleted from the selected studies as duplicates. The inclusion of review articles will give us a prospect to examine the synthesized evidence in existing reviews and reduce our effort to re-review of the individual studies. Besides that, multi country studies or trials that included findings from the South Asian countries will be included in our review as well to ensure the utmost findings are being captured related to COPD in South Asia. Throughout this process, three reviewers will be involved. The review will be conducted following a two-stage screening process: (1) title and abstract review and (2) full-text review. The first stage will involve the review of titles and abstracts by two reviewers to determine the studies eligibility based on the inclusion and exclusion criteria. The criteria will be tested on a sample of abstracts prior to beginning of the abstract review to ensure that they are robust enough to capture any articles that may relate to COPD in South Asia. All the titles and abstracts extracted from the three databases will be reviewed by these two reviewers independently. Any articles that are deemed appropriate by either or both of the reviewers will be included in the second stage of the selection process—a full-text review. During the full-text review, both reviewers will independently do a full-text screening of the articles selected at the first stage. Based on the review, identified articles will be included or excluded in this scoping review. Cohen’s κ statistic [26, 27] will be calculated at both the title and abstract review stage and at the full-article review stage to determine the inter-rater agreement. A difference of opinion can happen at any stage of review. Therefore, a third reviewer with the domain expertise will resolve any discordance between the two primary reviewers. The whole process will be done systemically with the help of web-based software known as “Rayyan QCRI,” developed by the Qatar Computing Research Institute (http://rayyan.qcri.org) [28].

### Step 4: charting the data

In this stage, a draft charting form will be developed in Microsoft Excel to aid the collection and sorting of crucial pieces of information from the selected articles. This process aims to produce a descriptive summary of the results that link to the concept and research questions of this scoping review. Characteristics to be extracted from the selected articles include standard information, such as the name of author/s, year of publication, type of publication, and study objectives, definition of COPD etc. (Additional file 3: Table S3 for the proposed draft charting form). In addition, information related to the research questions of this scoping review such as the prevalence of COPD, the risk factors of COPD, description of interventions, types of intervention (pharmacological/non-pharmacological intervention), and outcome of intervention will be extracted from the selected articles (Additional file 3). Information on interventions will be grouped into three categories depending on type, pharmacological, non-pharmacological, and a combination of pharmacological and non-pharmacological interventions. We will observe dyspnoea, functional/exercise capacity, frequency of acute exacerbations, health-related quality of life, hospitalisations and emergency department visits, lung function parameters, and all-cause mortality as outcome (Additional file 3). Each outcome will be grouped according to the types of outcome and, then will be presented in a table with the definition for each outcome type. In addition to that, the non-pharmacological interventions will be classified according to the Cochrane EPOC Review Group’s taxonomy [29]. Further revisions of the form will be done based on the review of the research team. The validity of the form will be pre-tested by the team members to ensure accurate information is collected from the articles. The finalized form will be used by two independent reviewers to extract the data from the included articles. A comparison of each reviewer’s abstracted data will be made and any discrepancies between them will be discussed with two primary reviewers and a third reviewer to ensure the coherence of opinion among the reviewers.

### Step 5: collating, summarizing, and reporting the results

**Fig. 2:**
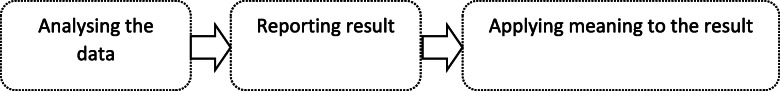
Steps of analyzing and reporting the result

Preferred Reporting Items for Systematic reviews and Meta-Analyses extension for Scoping Reviews (PRISMA-ScR) [30] will be used to present results from the search process. The PRISMA-ScR checklist will be adjusted by incorporating the elements that will derive from our research questions. To present our result in a rigorous manner, we will use the following three distinct stages proposed by Levac et al. [23] (Fig. 2).

Firstly, a descriptive numerical summary will be provided, stating the characteristics of the included articles, such as the total number of articles included, types of study design, year of publications, types of interventions, characteristics of the study populations, and countries where studies were conducted. Secondly, tables and figures will be used to present the results in an organized way, so that it satisfies the purposes of the research questions in the scoping review. Finally, the meaning of the study result will be elaborated under the light of research, policy, and practice, so that it will help us to write the recommendations based on the findings. The result of this scoping review will be presented in two separate manuscripts. The first will be based on research question one and two (prevalence and risk factors of COPD) and the second manuscript will be based on research question three (Interventions for COPD).

### Step 6: consultation

Though this step of Arksey and O’Malley’s methodological framework is an optional one, we will apply this step to identify the gaps in our literature review. The purpose of this consultation is to identify the literature that we have missed during step 2 “identifying relevant studies.” During the literature review, we will identify researchers from every country in South Asia who has a good track record in publishing literature or research and has experience on COPD research. We will try to contact them and send them the final list of the selected literature that we will compile during the identification of literature phase. Any suggestions for additional literature will be taken into consideration. Literature suggested by them will be included only if those meet the inclusion and exclusion criteria of the study.

### Additional step: quality assessment

This step aims to evaluate the quality of the studies which will be included in the scoping review. This quality assessment will give an added advantage to understand the meaning of the study result. However, the quality assessment will not be a part of the inclusion criteria that we mentioned earlier, and no study will be excluded based on this quality assessment. The quality assessment of the selected studies will be made using critical appraisal tools from The Joanna Briggs Institute. The Joanna Briggs Institute provides a separate tool to evaluate different study designs. Each tool has a set of questions to assess the studies [31]. By answering the questions, the reviewer will assess the studies. The quality appraisal process will be done by two reviewers independently, and like step 3 of the scoping review, we will calculate Kappa statistics [26, 27] to observe inter-rater reliability. The selected studies’ quality appraisal will be tabulated based on the questions of critical appraisal tools from the Joanna Briggs Institute. The reviewer will have three response options for each question; whether the study (1) met the criteria, (2) did not meet the criteria, or (3) if the answer of that selected question is clearly presented in the reviewed document or not.

## Discussion

To our knowledge, there has been no previous attempt to understand the scenario of COPD form a South Asian perspective. Part of the justification for using a scoping review method was to justify the inclusion of a wide range of sources of information (e.g., thesis, conference presentation, and reports) falling outside the typical article. However, this approach might also lead us to some limitations. Such as if an article with a multi-country study/trial did not provide country specific information, it will be difficult to extract country-specific information from the manuscript. In this regard, we will contact the respective authors of those manuscripts and try to collect country-specific information if the manuscript includes data related to specific South Asian countries. Additionally, some review articles that are not country- or region-specific in their title or abstract can be missed. These articles might include studies from South Asia. This problem could be minimized by doing a hand search of the review articles using Google and Google Scholar, and a backward search of key review articles (step 2). We are also anticipating the risk of publication bias if our scoping review fails to identify all available data on our topic, and subsequently, the description of information will be incomplete. The risk of publication bias will be mitigated by consulting researchers in the field of COPD (step 6) and implementing the hand search strategy of available resources (step 2).

### Ethical approval

Ethical approval will not be required to conduct this study since we are exploring publicly available material to answer the research questions of the scoping review. To facilitate the finding of the research article concerning COPD in South Asia, we will consult with researchers from South Asia, where we will not collect any personal information or any information that requires ethical approval. Furthermore, this review will provide an overview of the information that is available in the existing literature about COPD in South Asia.

## Conclusion

Through this scoping review, we will aim to give a complete picture of COPD in South Asia. Since this review will be covering key aspects of COPD, therefore, clinicians, policy-makers, and public health researchers will be benefitted from the outcome of the study. Furthermore, this proposed scoping review can act as a precursor to systematic review or meta-analysis. This scoping review can help reviewers to develop and confirm their priori inclusion criteria and the research questions of subsequent systematic reviews and meta-analysis by identifying available and relevant research papers. However, in order to offer a clear, transparent, and strong review, we developed this protocol so that further problems during the undertaking of the review can be avoided.

## Supplementary Information

**Additional file 1.** PRISMA-P 2015 checklist

**Additional file 2.** Proposed search term

**Additional file 3.** Table S3: Proposed draft charting form

## Data Availability

Not applicable

## Abbreviations

COPD: Chronic obstructive pulmonary disease
CRD: Chronic respiratory diseases
GOLD: Global Initiative for Obstructive Lung Disease
LMICs: Low- and middle-income countries
IAP: Indoor air pollution
NCDs: Non-communicable diseases
PCC: Population-concept-context
PICO: Population, Intervention, Comparator, and Outcome
MeSH: Medical subject headings
PRISMA-ScR: Preferred Reporting Items for Systematic reviews and Meta-Analyses extension for Scoping Reviews
GAC: Global Affairs Canada
SIDA: Swedish International Development Cooperation Agency
UKAID: Department for International Development-GOV.UK
