# Supplementary material for: Prevalence, risk factors, and interventions for chronic obstructive pulmonary disease in South Asia: a scoping review protocol
Source: Syst Rev. 2021 Jan 11;10:20. doi: 10.1186/s13643-020-01556-7 (PMC7798320; doi:10.1186/s13643-020-01556-7)
Supplement: Supplementary file 2 — Additional file 2. Proposed search term [file 13643_2020_1556_MOESM2_ESM.zip › Additional file_Proposed search term_No Track ChangeR5.docx]

**Additional file 2: Proposed search term**

| **Subject heading** | **Search terms used** |
| --- | --- |
| **Chronic Obstructive Pulmonary Disease** | (copd[MeSH Terms] OR coad[MeSH Terms] OR chronic obstructive pulmonary disease[MeSH Terms] OR chronic obstructive airway disease[MeSH Terms] OR chronic obstructive lung disease[MeSH Terms] OR chronic bronchitis[MeSH Terms] OR emphysema[MeSH Terms] OR copd OR coad OR chronic obstructive pulmonary disease OR chronic obstructive airway disease OR chronic obstructive lung disease OR chronic bronchitis OR emphysema) |
| **AND** | |
| **Prevalence** | (prevalence[MeSH Terms] OR frequency[MeSH Terms] OR epidemiology[MeSH Terms] OR prevalence OR frequency OR epidemiology |
| **OR** | |
| **Risk factor** | risk factors[MeSH Terms] OR determinant, epidemiologic[MeSH Terms] OR risk factors OR epidemiological determinant |
| **OR** | |
| **Intervention** | intervention study[MeSH Terms] OR randomized controlled trial[MeSH Terms] OR pilot studies[MeSH Terms] OR feasibility studies[MeSH Terms] OR community based intervention OR management OR control OR treatment OR strategy OR intervention study OR randomized controlled trial OR pilot studies OR feasibility studies |
| OR | |
| **Review** | review, systematic[MeSH Terms] OR meta analysis[MeSH Terms] OR meta analyses OR meta syntheses OR scoping review OR narrative review OR rapid review OR critical review OR integrative review OR systematic review OR meta analysis) |
| **AND** | |
| **South Asian countries** | (Afghanistan OR Bangladesh OR Bhutan OR India OR Maldives OR Nepal OR Pakistan OR Sri Lanka OR South Asia OR Asian OR Asia) |

**Full search strategy**

(copd[MeSH Terms] OR coad[MeSH Terms] OR chronic obstructive pulmonary disease[MeSH Terms] OR chronic obstructive airway disease[MeSH Terms] OR chronic obstructive lung disease[MeSH Terms] OR chronic bronchitis[MeSH Terms] OR emphysema[MeSH Terms] OR copd OR coad OR chronic obstructive pulmonary disease OR chronic obstructive airway disease OR chronic obstructive lung disease OR chronic bronchitis OR emphysema) AND (prevalence[MeSH Terms] OR frequency[MeSH Terms] OR epidemiology[MeSH Terms] OR prevalence OR frequency OR epidemiology OR risk factors[MeSH Terms] OR determinant, epidemiologic[MeSH Terms] OR risk factors OR epidemiological determinant OR intervention study[MeSH Terms] OR randomized controlled trial[MeSH Terms] OR pilot studies[MeSH Terms] OR feasibility studies[MeSH Terms] OR community based intervention OR management OR control OR treatment OR strategy OR intervention study OR randomized controlled trial OR pilot studies OR feasibility studies OR review, systematic[MeSH Terms] OR meta analysis[MeSH Terms] OR meta analyses OR meta syntheses OR scoping review OR narrative review OR rapid review OR critical review OR integrative review OR systematic review OR meta analysis) AND (Afghanistan OR Bangladesh OR Bhutan OR India OR Maldives OR Nepal OR Pakistan OR Sri Lanka OR South Asia OR Asian OR Asia)
