# Supplementary material for: Prevalence, risk factors, and interventions for chronic obstructive pulmonary disease in South Asia: a scoping review protocol
Source: Syst Rev. 2021 Jan 11;10:20. doi: 10.1186/s13643-020-01556-7 (PMC7798320; doi:10.1186/s13643-020-01556-7)
Supplement: Supplementary file 3 — Additional file 3. Table S3: Proposed draft charting form [file 13643_2020_1556_MOESM3_ESM.docx]

**Table S3: Proposed draft charting form**

| **Bibliographic Information** | **Aims and method of the study** | **Research Questions** | | |
| --- | --- | --- | --- | --- |
|  |  | **1^st^ Research Question** | **2^nd^ Research Question** | **3^rd^ Research Question** |
| Article title | Objectives of the study | Prevalence of COPD   1. By Sex 2. By different age group 3. By Socioeconomic status 4. By living region (urban vs rural) 5. By different occupation 6. By different level of education 7. By definition of COPD (Gold Criteria vs Lower limit of normal (LLN) threshold) 8. By smoking status (Current vs Former 9. By biomass burning status 10. By different stage of COPD) | Risk factors of COPD  a) The strength of the risk factors (OR or RR with 95% CI) of COPD such as   1. Male sex 2. Smoking status 3. Passive smoking status 4. Low educational level 5. Low BMI 6. Family history of respiratory disease 7. Allergy history 8. Respiratory infection during childhood 9. Recurrent respiratory infection 10. Occupational dust exposure 11. Biomass burning 12. Poor housing ventilation 13. Cooking frequently 14. Living around a polluted area 15. Living in a city or town vs living in the countryside 16. Doing physical labor work 17. Genetic risk factor.   b) Details of the statistical analysis (such as type of analysis and variables adjusted in the analysis). | Intervention for COPD  1. Type of Intervention   - Pharmacological Intervention - Non-Phramacological intervention   combination of pharmacological and non-pharmacological intervention.  Outcome of Intervention   - dyspnoea and - functional/exercise   capacity,   - frequency of acute exacerbations, - health-related   quality of life,   - hospitalisations - emergency department - visits) and - lung function parameters - All-cause mortality: Number of patients who died during the study.   2. Comparator and details of the intervention and control arm.  3. Duration of the intervention.  4. Details of the statistical analysis (such as type of analysis and variables adjusted in the analysis). |
| Publication year | Study setting |  |  |  |
| Author list | Study design |  |  |  |
| Type of Publication | Sample size and population |  |  |  |
|  | Definition of COPD |  |  |  |
